# Tick-Borne Infections: Results from a Prospective Observational Study from a Single Centre in Northern Italy

**DOI:** 10.3390/pathogens15070736

**Published:** 2026-07-14

**Authors:** Andrea Tedesco, Pietro Sponga, Giulia Bertoli, Andrea Matucci, Graziana Da Rold, Federica Obber, Lucia Moro, Chiara Piubelli, Francesca Perandin, Concetta Castilletti, Fabio Formenti, Cristina Mazzi, Salvatore Scarso, Dora Buonfrate, Federico Gobbi

**Affiliations:** 1 Department of Infectious, Tropical Diseases and Microbiology, IRCCS Sacro Cuore Don Calabria Hospital, Negrar di Valpolicella, 37024 Verona, Italy; andrea.tedesco@sacrocuore.it (A.T.); giulia.bertoli@sacrocuore.it (G.B.); andrea.matucci@sacrocuore.it (A.M.); lucia.moro@sacrocuore.it (L.M.); chiara.piubelli@sacrocuore.it (C.P.); francesca.perandin@sacrocuore.it (F.P.); concetta.castilletti@sacrocuore.it (C.C.); fabio.formenti@sacrocuore.it (F.F.); cristina.mazzi@sacrocuore.it (C.M.); salvatore.scarso@sacrocuore.it (S.S.); federico.gobbi@sacrocuore.it (F.G.); 2 Department of Infectious and Tropical Diseases, Careggi University Hospital, 50134 Florence, Italy; pietro.sponga@gmail.com; 3 Unit of Ecopathology Wildelife Specialistic Center-SCT2-Belluno, Istituto Zooprofilattico Sperimentale delle Venezie (IZSVe), 32100 Belluno, Italy; gdarold@izsvenezie.it (G.D.R.); fobber@izsvenezie.it (F.O.); 4 Department of Clinical and Experimental Sciences, University of Brescia, 25123 Brescia, Italy

**Keywords:** tick-borne diseases, vector-borne diseases, TBE, Lyme borreliosis, borreliosis, SENLAT

## Abstract

Background: Data on tick-borne diseases (TBDs) in Italy are fragmented. This study aimed to describe tick-borne pathogens (TBPs) and TBDs observed in a hospital in Italy and to assess the correlation between TBPs and human disease. Methods: A prospective observational study was conducted in a single hospital between March 2024 and January 2025. Inclusion criteria were: individuals ≥ 8 years of age and consent to study participation. Data and blood samples were collected at baseline (T0), and 12–18 weeks later (T1). Ticks collected at T0 were pooled and analyzed by real-time PCR. Serological and molecular tests were used for TBD diagnosis. Results: Ninety-three individuals (median age 39 years; 52.7% female) were enrolled. The pathogens most frequently detected in the tick pools were *Rickettsia* spp. (15.1%) and *Borrelia* spp. (7.5%). Sixteen participants (17.2%) developed TBD-compatible symptoms, but TBD was confirmed in only four cases: Lyme borreliosis (n = 2), scalp eschar and neck lymphadenopathy after tick bite (SENLAT, n = 1), and tick-borne encephalitis (TBE, n = 1). Concordance between TBP detection and the corresponding TBD development in humans was observed in only two cases (2.1%). Conclusions: The low concordance between pathogens detected in participant-level tick pools and confirmed human tick-borne disease supports the limited clinical value of tick testing in this setting.

## 1. Introduction

Ticks are the second most important arthropod vector for human diseases after mosquitoes [1,2]. Present on every continent, including Antarctica, ticks can carry viral, bacterial and parasitic pathogens, which pose risks for both animals and humans [1,2,3]. The geographical distribution of ticks and tick-borne diseases (TBDs) is rapidly evolving across Europe [4,5], with expansion into areas previously classified as non-endemic [6]. Several factors contribute to the spread of TBDs, with particular emphasis on global warming, which can influence the vector life cycle [7]. Additional drivers include the accelerated destruction of natural habitats and the expansion of urban settlements up to the edge of wild areas [7]. The combined effects of these factors have been especially evident in the expansion of Lyme borreliosis and tick-borne encephalitis (TBE) [5].

While Lyme borreliosis remains the most widely recognized of these conditions, other serious illnesses—such as tick-borne encephalitis (TBE), anaplasmosis, and babesiosis—pose distinct medical challenges. TBDs often have similar signs and symptoms, which can also mimic a generic viral illness in early phases. Thus, monitoring the local circulation of TBPs and their transmission dynamics is essential for timely diagnosis [3].

Published literature on TBDs in Italy is fragmented, reporting tick-borne pathogen (TBP) prevalence, clinical characteristics, or tick distribution at the provincial level [8,9,10]. The fragmented state of knowledge and the heterogeneous methodologies employed to gather the data—veterinary and/or human medical perspectives, retrospective and prospective study designs—hinder the analysis of TBP-TBD dynamics and their evolution. The interpretation of surveillance data is further limited by the fact that many of these diseases are not subject to mandatory reporting, likely leading to underestimation of their actual prevalence [11,12,13]. This limitation holds true both for diseases already recognized as endemic in Italy (e.g., TBE, LD), and for those that may emerge, such as Crimean–Congo hemorrhagic fever [14].

Although data on TBP prevalence in vectors is valuable, it provides limited insight into disease transmission and actual penetration in the human host. A more complete assessment requires surveillance systems or studies targeting both TBP and TBD; evidence on this has been recently reported in a retrospective study carried out in France [15].

The aim of this study was to describe the epidemiology of TBPs detected in both patients and ticks analyzed at a single hospital in Northern Italy. The primary objective was to estimate the proportion of TBPs leading to TBDs following a tick bite. Secondary objectives were to: (i) estimate the proportion of participant-level tick pools testing positive for at least one pathogen; (ii) estimate the proportion of TBPs transmitted to humans following a tick bite; and (iii) identify emerging TBPs. An exploratory objective was to quantify the number of individuals who developed symptoms after a tick bite but remained undiagnosed.

## 2. Materials and Methods

This study was an observational prospective study carried out at the IRCCS Sacro Cuore Don Calabria, Negrar, Verona, Italy, between March 2024 and January 2025.

### 2.1. Participants

The population of Verona Province was informed about the study through public awareness campaigns. Participation was offered to all individuals eight years of age or older who presented to the hospital emergency room for a tick bite from the 1 March 2024 to the 31 January 2025. Inclusion criteria were: (i) available tick(s), already collected and delivered by the participant, or extracted with tweezers by a medical doctor at the emergency room; (ii) signed informed consent for study participation. Informed consent signed by parents or a guardian was sought for the participation of all individuals below 18 years of age; moreover, the child’s consent was sought for participants from 12 to 17 years of age. Children under eight years of age were excluded to avoid blood sampling in an age group considered at lower risk of tick bite and with reduced compliance to the study procedures.

A baseline visit (T0) was scheduled between seven and ten days from the tick bite. T0 included data collection using a study case report form (CRF) available in the REDCap database and blood sampling for baseline serology tests. Data collection included the duration of tick attachment (as reported by the participant), the geographical area where the tick bite occurred, previous episodes of tick bite, and vaccination history for TBE. The participants were instructed to contact the study investigators if any signs/symptoms emerged that were possibly evocative of TBD, provided in a list prepared for study purposes. In such cases, participants were invited to attend an unscheduled visit (TU) with additional blood sampling for clinical and laboratory evaluation (which included molecular tests). All participants, including those who were diagnosed with a TBD at a TU, were then invited to a follow-up visit (T1) scheduled 12–18 weeks after the tick bite. At T1, blood sampling was performed and additional data were recorded, including antibiotic treatments for other conditions unrelated to the tick bite. All collected biological samples were stored at −80 °C in Tropica Biobank (BBMRI-eric ID: IT_1605519998080235) until further processing. Participants diagnosed with any TBDs received treatment in accordance with standard clinical practice.

### 2.2. Tick Identification and Processing

All collected ticks were preserved at −80 °C until species identification was done by trained microscopists using morphological keys. Briefly, ticks were morphologically identified at the lowest achievable taxonomic level, ranging from family to species, using dichotomous keys [16,17]. Identification was performed according to state of preservation and anatomical integrity. The total nucleic acids from ticks were isolated using a thermo-enzymatic extraction protocol with the RNAGem^®^ Kit (Microgembio, Southampton, UK), according to the manufacturer’s instructions for complex tissue extraction without final DNase I treatment. The nucleic acid extracts were subjected to molecular tests, as indicated in the following paragraph. In order to contain costs and speed up the analyses, all larvae and nymphs retrieved from the same participant were pooled in the same vial and processed together for nucleic acid extraction, while adults (if more than one for the same participant) were pooled and analyzed separately. Eventually, we considered each pool composed of all the ticks (irrespective of stage) removed from a participant and reported all TBPs detected per pool.

### 2.3. Molecular Tests

Nucleic acid extraction from human blood samples was performed on an automated sample DNA/RNA purification instrument (EZ1 Advanced XL, Qiagen, Hilden, Germany) using the DSP virus kit (EZ1 DSP Virus Kit, Qiagen, Hilden, Germany). TBPs target nucleic acid amplification was performed on nucleic acid extracts from whole blood and ticks using CE-IVD real-time PCR kits as previously described [18]. Briefly, the following kits were used: the alphaCube kits (Mikrogen Diagnostik, Neuried, Germany) targeting TBEV, *Borrelia burgdorferi* s.l./*Rickettsia* spp. and *Ehrlichia* spp. (including *Anaplasma phagocytophilum*); and the Viasure Tick Borne Disease Multiplex kit (Certest Biotec, Zaragoza, Spain) targeting TBEV, *B. burgdorferi* s.l./*Borrelia miyamotoi*/*Borrelia hermsii*, *Rickettsia* spp., *Anaplasma phagocytophilum*, *Ehrlichia chaffeensis*/*Ehrlichia muris*, *Babesia microti*/*Babesia divergens* and *Coxiella burnetii.* Both kits were used according to the manufacturer’s instructions on a CFX96 Touch Real-Time PCR Detection System (Bio-Rad, Milan, Italy). Results obtained from one or both kits were reported. *Borrelia* species were further characterized using species-specific TaqMan real-time PCR assays, enabling discrimination among *B. miyamotoi*, *B. afzelii*, *B. garinii*, and *B. burgdorferi* sensu stricto as previously indicated [19]. In order to identify the species, samples positive for *Rickettsia* spp. and *Babesia* spp. were further analyzed using specific endpoint PCRs followed by Sanger sequencing as previously indicated [10]. Sequences were aligned and compared with those available in GenBank using the Basic Local Alignment Search Tool (BLAST+2.17.0 http://blast.ncbi.nlm.nih.gov/Blast.cgi, last accessed on 24 March 2025). When possible, species identification was assigned when sequence identity was ≥99% (GenBank accession number ID: OR148326 for *Rickettsia slovaca*; EU883092 for *Rickettsia monacensis*; KJ663750 for *Rickettsia helvetica*; KU351826 for *Babesia capreoli*).

### 2.4. Serological Tests

Serum samples collected at T0 and TU/T1 for each participant were analyzed on the same run to observe possible seroconversion. Commercial immunofluorescence assays (IFA) were used for TBEV (Mosaic Flavivirus 1, Euroimmun AG, Lübeck, Germany), and *Rickettsia conorii*, *R. rickettsii* and *R. typhi* (Fuller Laboratories, Fullerton, CA, USA). Screening for *B. burgdorferi* sensu lato (s.l.) IgG and IgM was performed by commercial ELISA (*Borrelia* plus VlsE IgG ELISA, and *Borrelia* select IgM ELISA, Euroimmun AG, Lübeck. Germany), and a commercial immunoblot (recomLine *Borrelia* IgG and IgM, Mikrogen Diagnostik, Neuried, Germany) was used for confirmation.

*B. burgdorferi* s.l. seroconversion was defined as a change in IgG status from negative to positive between T0 and T1, based on the combined results of the IgG ELISA and immunoblot assays.

### 2.5. Statistical Analysis

A convenience sample including all participants (and related ticks) who presented during the study period was used for analysis. Variables were described using descriptive statistics. Categorical values were summarized using absolute frequencies and percentages, while continuous variables were summarized as median and interquartile range (Q1, Q3) and range (Max–Min). All statistical tests were performed using a 5% significance level, unless otherwise stated. R statistical software, version 4.5.1, was used for analysis.

## 3. Results

### 3.1. Participants

**Figure 1:**
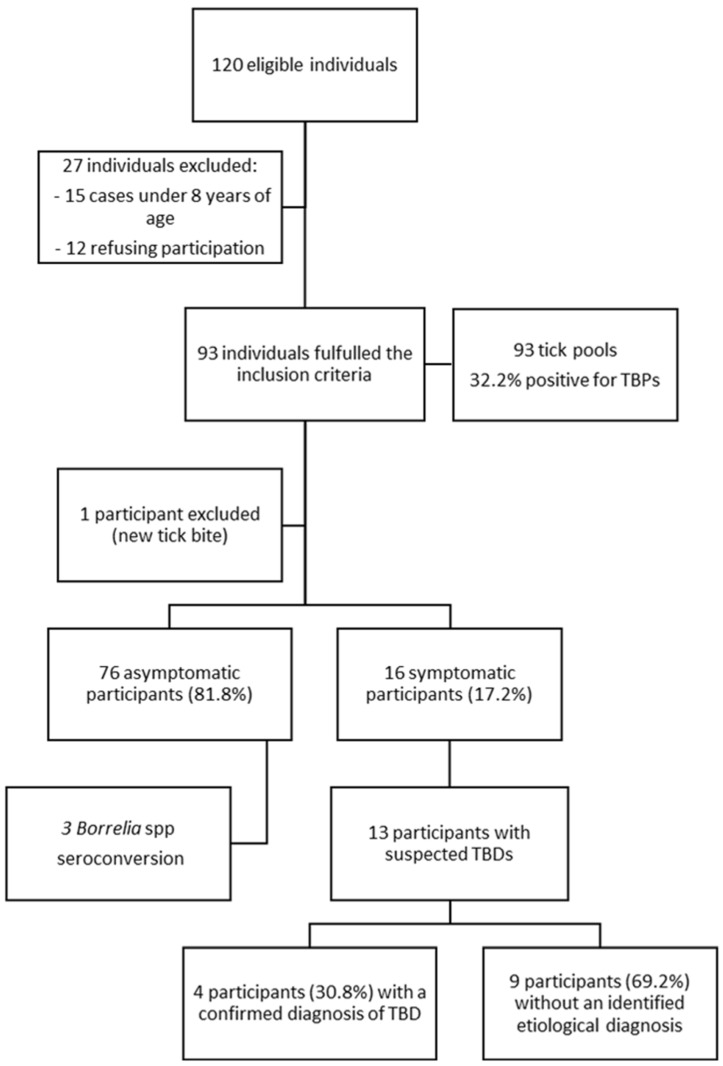
Flow diagram of study participants. The figure depicts the number of patients included in each phase of the study.

A total of 120 individuals presented to our hospital for tick bites, and 93 of them were enrolled in our study (Figure 1). Of these 93 participants, 49 (52.7%) were females and 44 (47.3%) males. The median age was 39 years (range 8–84; Q1, Q3, 14, 58). No participant was lost to follow-up; they all presented either to T1 or TU.

**Figure 2:**
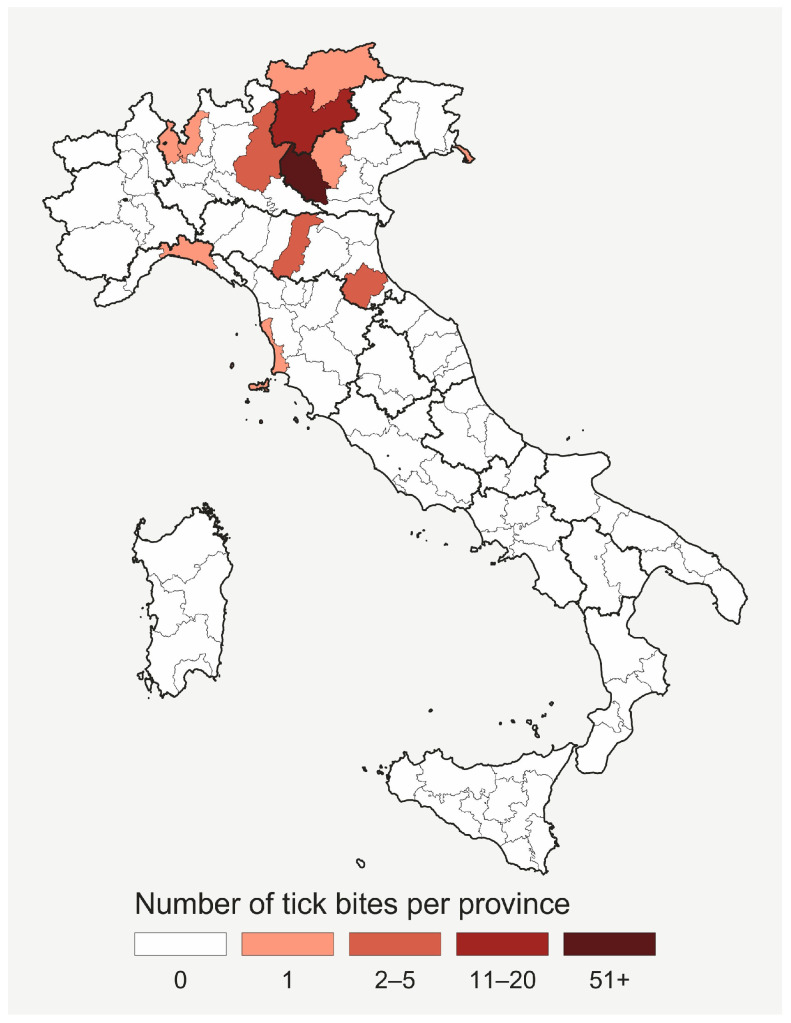
Geographical distribution (at province level) of the areas where tick bites occurred. The geospatial map was created in the R environment using the sf package [20]. The administrative boundaries of the Italian provinces were obtained from the Openpolis geojson-italy dataset (https://github.com/openpolis/geojson-italy, accessed on 1 July 2021, second release), which is publicly available on GitHub and distributed under a CC BY 4.0 license.

Most participants (65 of 93, 71.4%) reported that the bite had occurred in the Veneto region, followed by 12 (13.2%) in Trentino Alto-Adige, seven (7.7%) in Emilia-Romagna, four (4.4%) in Lombardy, and one each in Friuli Venezia Giulia, Liguria and Tuscany. Two bites occurred abroad (Figure 2).

Twelve out of 93 participants (12.9%) reported a history of previous TBE vaccination, while two participants reported a previous diagnosis of Lyme borreliosis. A previous episode of tick bite was reported by 27 (29%) participants.

### 3.2. Characteristics of Ticks and Tick Pools

In 77/93 (82.8%) cases, the participant presented with one tick; in six (6.5%) cases, with two ticks; and in 10/93 (10.8%) cases, with three or more ticks.

A total of 143 ticks were obtained from the participants. The cohort of ticks collected over the study period mostly overlaps with the one described previously [18]. Among the 93 tick pools included in the study, 90 (96.8%) were morphologically classified as *Ixodes* spp. The remaining samples included one *Dermacentor marginatus*, one *Aponomma* spp. (originating from Malawi), and one *Hyalomma marginatum*. Tick pools could contain ticks of different developmental stages. Most of them (64 out of 93, 68.8%) contained nymphs, followed by adults (23/93, 24.7%), and larvae (4/93, 4.3%). Two tick pools were too damaged to allow reliable determination of their developmental stage.

Overall, 30 tick pools (32.2%) were positive for at least one pathogen. Specifically, 23/93 (24.7%) pools were positive for one pathogen, and seven (7.5%) were positive for two pathogens. *Rickettsia* spp. were the infective agents most frequently detected (15.1%, 14/93), followed by *Borrelia* spp. (7.5%, 7/93), *Ehrlichia* spp. (7.5%, 7/93), and *Anaplasma phagocytophilum*. (5.4%, 5/93). *Babesia* spp. and TBEV were detected in 2 (2.2%) and 1 (1.1%) pools, respectively. Among the 14 *Rickettsia* spp. identified, 7 were *R. helvetica*, 3 *R. monacensis*, 1 *R. slovaca*, and 3 could not be typed to the species level. Real-time PCR detected the presence of 3 *B. afzelii*, 2 *B. burgdorferi* sensu stricto, and 2 *B. miyamotoi* among *Borrelia* spp. Only one *Babesia* was identified by sequencing as *B. capreoli;* the other was not typeable.

### 3.3. Clinical Syndromes and Associations Between Tick-Borne Pathogens and Disease

Seventeen out of 93 participants (18.2%) presented to a TU. One was excluded because she reported a new tick bite. The remaining 16 (17.2%) reported the following symptoms: headache in eight out of the sixteen participants (50%), fever in six cases (35.3%), myalgia in four cases (25%), arthralgia and paresthesia in three (19%) cases, asthenia in two cases, and subjective cognitive dysfunction (reported as “mental fog”) in one case. Three participants presented cutaneous manifestations: erythema migrans was observed in two cases and an eschar in one case.

All 16 patients received screening tests as described in the Methods. Eventually, four participants out of the 16 reporting symptoms were confirmed as TBDs (25%), and a correlation between the TBPs detected in the tick pools and the TBDs was confirmed in two participants (Table 1). The median number of hours of attachment of the tick to the human host was 24 (min 0, max 552); the duration of the tick attachment did not seem to correlate with TBP transmission, nor with the emergence of symptoms.

Data correlating tick-borne pathogens and tick-borne diseases are summarized in Table 1.

### 3.4. TBD Cases

An eight-year-old girl developed a scalp eschar and painful retroauricular lymphadenopathy, without systemic symptoms, four days after a tick bite. The tick was identified as an adult female *Dermacentor marginatus* and tested positive for *R. slovaca* by sequencing analysis. Both the tick and the eschar swab yielded positive results for *Rickettsia* spp. by real-time PCR. Serological tests were negative for IgG but positive for IgM, consistent with an acute infection, while whole blood real-time PCR was negative for *Rickettsia* spp. The patient was successfully treated with a six-day course of azithromycin. At the three-month follow-up, the eschar had healed completely. No serological seroconversion was observed.

Two participants presented with erythema migrans associated with Lyme borreliosis. Both patients were bitten by an adult female *Ixodes ricinus*. One tick pool tested positive by real-time PCR for both *B. burgdorferi* and untypeable *Rickettsia* spp.; the other tested positive for both *R. helvetica* and *B. capreoli*. The two patients were treated with a ten-day course of doxycycline. Neither the serological nor the molecular tests on the patients confirmed *Borrelia* infection.

Another participant presented with worsening asthenia, limiting daily life activities. Clinical examination and lab tests excluded any cardio-respiratory signs. The participant tested positive for TBEV in whole blood extract and was strictly monitored for possible neurological worsening. The symptoms remained mild and slowly cleared during the follow-up period. The patient never seroconverted with TBEV.

### 3.5. Participants Without a Definite Diagnosis of TBD

Three of the sixteen participants with symptoms were diagnosed with other conditions, namely SARS-CoV-2, Parvovirus B19 (HPV-B19) infections, and reactivation of multiple sclerosis. For nine participants, it was not possible to identify an etiological cause. Among these nine participants, the most frequent symptom was headache alone; in one case, it was associated with a flu-like syndrome, and in another with fever. Seroconversion was not observed in these patients for any pathogen in the study. Of note, participants 5 and 13 presented with persistent symptoms and were hospitalized. Participant 5 underwent lumbar puncture, which was negative for all tested pathogens. Lumbar puncture was not performed in participant 13 due to tonsillar herniation. However, due to positive IgM for *B. burgdorferi* s.l., and in the absence of any other positive test for other diseases, participant 13 was treated for suspected neuroborreliosis with intravenous ceftriaxone 2 g/day for 4 days, followed by oral doxycycline 100 mg twice a day for 7 days. No seroconversion was observed at follow-up; however, this could be expected due to the antibiotic treatment. Overall, treatment was deemed justified considering the possible harm of untreated neuroborreliosis.

Separately, three out of the 76 asymptomatic patients (3.9% of the whole cohort) had *B. burgdorferi* s.l. seroconversion. All these patients were treated with a 14-day course of doxycycline 100 mg twice a day.

## 4. Discussion

In our study, concordance between pathogens detected in tick pools and the final diagnosis of TBD was low (2/93, 2.1%). This finding supports current guidelines [21,22] that recommend refraining from testing ticks supplied by patients for clinical management purposes. Moreover, while TBD-related symptoms were reported by 14% (13/93) of participants, only four of them (4.3% of the whole cohort) were eventually diagnosed with TBD. Excluding pathognomonic symptoms for Lyme borreliosis and SENLAT, the other symptoms were nonspecific. Although clinical manifestations may have had a non-tick-related origin, limitations of the available diagnostic tests cannot entirely exclude that some TBDs were undetected.

In a cohort of patients exposed to tick bites in France between 2014 and 2021 [15], the proportion of TBD diagnoses (26 of 217, 12%) was considerably larger than that found in this study. The discrepancy might be due to a different epidemiology and study design (retrospective versus prospective). Notably, no participants were lost to follow-up in our cohort, and all were instructed to report any TBD-compatible symptoms (including headache, asthenia); therefore, the likelihood of having missed TBD cases is low.

In Italy, SENLAT has been mainly described in central regions [23], although surveillance studies conducted in northern areas confirm the presence of tick vectors and rickettsial pathogens related to this syndrome. However, incidence rates in Italy, and in particular in northern regions, remain low [9]. This could be due to a limited number of cases, as well as to poor clinical awareness and access to molecular diagnostics. A case series described ten SENLAT cases observed over seven years in Tuscany. Different from our case, which occurred in a child, they diagnosed SENLAT in women [23]. Another difference is that we could achieve laboratory confirmation [23], while their diagnosis of SENLAT was made on a clinical basis.

In our cohort, two participants presented with single erythema migrans, leading to a clinical diagnosis of early-stage Lyme borreliosis. Of note, laboratory results from humans and ticks were not in line with this diagnosis. Although erythema migrans is related to borreliosis [24], little is known about the pattern of serological response in co-infection with *Rickettsia* spp. and *Borrelia burgdorferi* s.l. [25]. Moreover, a Norwegian research group questioned whether all erythema migrans cases are *Borrelia* spp.-related or may instead be attributed to other microorganisms, reporting a few cases of erythema migrans caused by the genus *Neoehrlichia* [26]. More insights on this topic are necessary to give a definitive answer.

Only one case of TBE was identified by a molecular test during the initial phase of symptoms, without neurological progression, and with negative TBEV PCR from the tick pool. This result may be influenced by RNA degradation during arthropod processing and storage before testing [27].

Seroconversion with *B. burgforferi* s.l. (with negative molecular tests) was observed in three asymptomatic participants, raising the question regarding optimal clinical management. IDSA guidelines do not recommend treatment [21] for patients with positive serology in the absence of symptoms based on data showing that these patients are less likely to develop Lyme borreliosis compared to those with erythema migrans. However, most data come from seroprevalence studies [28], and less evidence is available concerning individuals with documented seroconversion; therefore, we decided to prescribe a regimen of doxycycline. Follow-up of these patients focused on the development of symptoms, aiming to reduce uncertainty in clinical management of individuals with seroconversion, for whom the risk of developing a TBD is unclear [21]. These cases also highlight that testing individuals with no clear clinical indication should be done with caution, as it may lead to possible overtreatment.

In Italy, official data on TBD epidemiology are presumably underestimated due to limited awareness among healthcare professionals, the absence of an effective surveillance system, and diagnostic challenges [29,30]. A retrospective study analyzed discharge records between 2017 and 2022 from emergency departments and hospital admissions in Azienda ULSS8 Berica, in our same region (Veneto) [31]. Total cases of TBD, Lyme borreliosis and TBE were 143, 31 and 31, respectively. Both TBD-related visits in the emergency departments and TBE hospitalizations had a peak in 2022 (29 and 11 cases, respectively), while Lyme borreliosis had a peak in 2019 (8 cases) and the lowest number of cases in 2022 [31]. While this type of study may be useful for identifying high-risk areas and monitoring the epidemiology of the most severe clinical presentations, it does not allow for evaluating the emergence of other TBDs. For this purpose, active surveillance involving community engagement currently represents the only viable approach.

After a tick bite, nonspecific symptoms such as fatigue, headache, myalgia, arthralgia, and neurocognitive complaints can develop, without laboratory evidence for a specific tick-borne infection. In these cases, management is uncertain [32,33]. It should be considered that many diagnostic gaps may hamper the achievement of TBD diagnosis, including the blunting of seroconversion following antibiotic treatment [21], the unreliability of serological assays [21,34], and the lack of testing for emerging pathogens [34]. Moreover, a substantial proportion of individuals do not recall being bitten by a tick. These factors highlight the need to maintain a high level of clinical suspicion during the spring and summer seasons [35], as negative diagnostic results do not exclude tick-borne infection, and clinical evaluation with appropriate testing remains essential for patient management.

In our cohort, the duration of tick attachment did not appear to be associated with TBP transmission nor with the emergence of symptoms. However, these findings should be interpreted with caution due to the substantial uncertainty of many patients reporting this information.

This study has some limitations. First, a convenience sample of individuals presenting during the study period was used; the number of participants and ticks included may limit the study power and the generalizability of the findings. Nevertheless, this is one of the largest studies to date analyzing both ticks and humans in Italy. Another limitation is that the diagnostic capacity was limited to available tests; hence, some TBD cases might have been missed because they could not be targeted by the assays used. However, it should be noted that none of the participants reporting symptoms had clinical progression/worsening; hence, from the clinical point of view, this is probably not relevant.

## 5. Conclusions

In conclusion, our findings support the limited clinical value of tick testing in this setting. The majority of suspected TBDs were not confirmed by laboratory tests. A TBD diagnosis was established in 4.3% of participants, but a correlation between TBPs and TBDs was confirmed in only two cases (2.1%). Reassuring patients who present after a tick bite and recommending follow-up, including appropriate testing based on the onset of symptoms, probably remains the most appropriate clinical approach. Further studies are needed to better define the diagnosis of tick-borne infection-related syndromes.

## Figures and Tables

**Table 1:** Clinical characteristics of participants reporting symptoms and related pathogens found in the tick pools.

Id/Sex/Age (years)	Pathogen(s) Found in Related Tick Pools	Clinical Manifestations	Diagnosis	Therapy	Agreement between Pathogen(s) in Tick Pool and Diagnosis
1/F/8	*Rickettsia slovaca*	Scalp eschar and cervical lymphadenopathy	SENLAT (molecular diagnosis)	6-day azithromycin	Yes
2/M/15	*Rickettsia* spp. *Borrelia* spp.	Erythema migrans, rash	Lyme borreliosis (clinical diagnosis)	10-day doxycycline	Yes
3/F/61	*Rickettsia* spp. *Babesia* spp.	Erythema migrans	Lyme borreliosis (clinical diagnosis)	10-day doxycycline	No
4/F/31	None	Asthenia, confusion, dyspnoea, hearing dullness	TBE (molecular diagnosis)	Symptomatic treatment	No
5/F/73	*Borrelia* spp.	Headache	None	Symptomatic treatment	No
6/F/37	*Anaplasma* spp.	Flu-like syndrome	None	None	No
7/F/11	None	Headache	None	None	No
8/M/61	None	Headache	None	None	No
9/F/9	None	Recurrent fever	None	None	No
10/M/76	None	Asthenia, myalgia	None	None	No
11/M/14	None	Flu-like syndrome, headache	None	None	No
12/F/22	None	Fever, headache	None	None	No
13/F/59	None	Headache	None	4-day ceftriaxone and 7-day doxycycline	No

## Data Availability

The raw data supporting the findings of this study are available in Zenodo: https://zenodo.org/records/17520046 accessed on 16 June 2026.

## Abbreviations

The following abbreviations are used in this manuscript:

TBD	Tick-borne disease
TBP	Tick-borne pathogen
TBE	Tick-borne encephalitis
SENLAT	Scalp eschar and neck lymphadenopathy after tick bite
